# Provision of Small-Quantity Lipid-Based Nutrient Supplements Increases Plasma Selenium Concentration in Pregnant Women in Malawi: A Secondary Outcome of a Randomized Controlled Trial

**DOI:** 10.1093/cdn/nzac013

**Published:** 2022-03-07

**Authors:** Marjorie J Haskell, Kenneth Maleta, Charles D Arnold, Josh M Jorgensen, Yue-Mei Fan, Ulla Ashorn, Andrew Matchado, Nagendra K Monangi, Ge Zhang, Huan Xu, Elizabeth Belling, Julio Landero, Joanne Chappell, Louis J Muglia, Mikko Hallman, Per Ashorn, Kathryn G Dewey

**Affiliations:** Institute for Global Nutrition, University of California, Davis, Davis, CA, USA; Department of Nutrition, University of California, Davis, Davis, CA, USA; Department of Public Health, School of Public Health and Family Medicine, College of Medicine, University of Malawi, Blantyre, Malawi; Institute for Global Nutrition, University of California, Davis, Davis, CA, USA; Department of Nutrition, University of California, Davis, Davis, CA, USA; Institute for Global Nutrition, University of California, Davis, Davis, CA, USA; Department of Nutrition, University of California, Davis, Davis, CA, USA; Center for Child, Adolescent and Maternal Health Research, Faculty of Medicine and Health Technology, Tampere University, Tampere, Finland; Center for Child, Adolescent and Maternal Health Research, Faculty of Medicine and Health Technology, Tampere University, Tampere, Finland; Institute for Global Nutrition, University of California, Davis, Davis, CA, USA; Division of Neonatology, Perinatal Institute, Cincinnati Children's Hospital Medical Center, Cincinnati, OH, USA; Department of Pediatrics, University of Cincinnati College of Medicine, Cincinnati, OH, USA; Department of Pediatrics, University of Cincinnati College of Medicine, Cincinnati, OH, USA; Division of Human Genetics, Cincinnati Children's Hospital Medical Center, Cincinnati, OH, USA; Center for Prevention of Preterm Birth, Perinatal Institute, Cincinnati Children's Hospital Medical Center and March of Dimes Prematurity Research Center Ohio Collaborative, Cincinnati, OH, USA; Division of Human Genetics, Cincinnati Children's Hospital Medical Center, Cincinnati, OH, USA; Center for Prevention of Preterm Birth, Perinatal Institute, Cincinnati Children's Hospital Medical Center and March of Dimes Prematurity Research Center Ohio Collaborative, Cincinnati, OH, USA; Division of Human Genetics, Cincinnati Children's Hospital Medical Center, Cincinnati, OH, USA; Center for Prevention of Preterm Birth, Perinatal Institute, Cincinnati Children's Hospital Medical Center and March of Dimes Prematurity Research Center Ohio Collaborative, Cincinnati, OH, USA; Department of Chemistry, University of Cincinnati, Cincinnati, OH, USA; Division of Human Genetics, Cincinnati Children's Hospital Medical Center, Cincinnati, OH, USA; Center for Prevention of Preterm Birth, Perinatal Institute, Cincinnati Children's Hospital Medical Center and March of Dimes Prematurity Research Center Ohio Collaborative, Cincinnati, OH, USA; Department of Pediatrics, University of Cincinnati College of Medicine, Cincinnati, OH, USA; Division of Human Genetics, Cincinnati Children's Hospital Medical Center, Cincinnati, OH, USA; Burroughs Wellcome Fund, Research Triangle Park, NC, USA; Medical Research Centre Oulu, PEDEGO Research Unit, University of Oulu, Oulu, Pohjois-Pohjanmaa, Finland; Center for Child, Adolescent and Maternal Health Research, Faculty of Medicine and Health Technology, Tampere University, Tampere, Finland; Department of Paediatrics, Tampere University Hospital, Tampere, Finland; Institute for Global Nutrition, University of California, Davis, Davis, CA, USA; Department of Nutrition, University of California, Davis, Davis, CA, USA

**Keywords:** selenium status, plasma, lipid-based nutrient supplements, pregnancy, Malawi

## Abstract

**Background:**

Pregnant women in Malawi are at risk of selenium deficiency, which can have adverse effects on pregnancy outcomes. Interventions for improving selenium status are needed.

**Objectives:**

To assess the effect of provision of small-quantity lipid-based nutrient supplements (SQ-LNSs) to Malawian women during pregnancy on their plasma selenium concentrations at 36 wk of gestation.

**Methods:**

Pregnant women (≤20 wk of gestation) were randomly assigned to receive daily either: *1*) iron and folic acid (IFA); *2*) multiple micronutrients (MMN; 130 µg selenium per capsule); or *3*) SQ-LNS (130 µg selenium/20 g). Plasma selenium concentrations were measured by inductively coupled plasma mass spectrometry at baseline and after ≥16 wk of intervention (at 36 wk of gestation) and compared by intervention group.

**Results:**

At 36 wk of gestation, median (quartile 1, quartile 3) plasma selenium concentrations (micromoles per liter) were 0.96 (0.73, 1.23), 0.94 (0.78, 1.18), and 1.01 (0.85, 1.28) in the IFA, MMN, and SQ-LNS groups, respectively. Geometric mean (GM) plasma selenium concentration was 5.4% (95% CI: 1.8%, 9.0%) higher in the SQ-LNS group than in the MMN group and tended to be higher than in the IFA group (+4.2%; 95% CI: 1.0%, 7.8%). The prevalence of adjusted plasma selenium concentrations <1 µmol/L was 55.1%, 57.8%, and 47.3% in the IFA, MMN, and SQ-LNS groups, respectively; it was lower in the SQ-LNS group than in the MMN group, OR = 0.44 (95% CI: 0.24, 0.83), and tended to be lower than in the IFA group, OR = 0.54 (95% CI: 0.29, 1.03). There was a significant interaction between baseline plasma selenium concentration and intervention group (*P *= 0.003). In the lowest tertile of baseline selenium concentrations, GM plasma selenium concentration was higher, and the prevalence of low values was lower in the SQ-LNS group compared with the MMN and IFA groups at 36 wk of gestation (*P *≤ 0.007).

**Conclusions:**

Provision of SQ-LNS containing selenium to pregnant women can be an effective strategy for improving their selenium status.

This trial was registered at clinicaltrials.gov (identifier: NCT01239693).

## Teaser

Provision of small-quantity lipid-based nutrient supplements containing 130 µg/d selenium to pregnant Malawian women for ∼16 wk increased plasma selenium concentrations in late pregnancy.

## Introduction

Selenium is an essential micronutrient required for reproduction, thyroxine metabolism, DNA synthesis, immune function, and protection from oxidative stress (1). Maternal selenium deficiency has been associated with adverse pregnancy outcomes, including preterm birth and preeclampsia (2–5). Intrauterine infection and an excessive inflammatory response are associated with adverse pregnancy outcomes, and the risk of these conditions can be higher in selenium-deficient women (2). Selenoproteins are involved in the immune response, and selenium-containing enzymes downregulate proinflammatory genes and thus can reduce the adverse effects of inflammation on pregnancy outcomes (2, 6, 7). A recent meta-analysis of 17 cohorts of pregnant women from geographically diverse areas showed that maternal selenium concentrations were significantly associated with preterm birth and gestational duration in some cohorts; however, the association was not generalizable across all the cohorts (8). Significant associations were observed between maternal selenium concentrations and preterm birth in women in the United Kingdom and Tanzania, and the most significant associations were observed in women in Malawi. No associations were observed for the other cohorts (8). In other studies, maternal serum selenium concentrations were also associated with preterm birth in Dutch women in the lowest quartile of serum selenium concentrations (2), and in selenium-deficient HIV-positive Nigerian women (5). In a recent study of Indonesian women, maternal serum selenium concentrations at the time of delivery were not associated with preterm birth; however, selenium concentrations of placenta and cord blood were lower in women with preterm deliveries compared with women who delivered at term (9).

The prevalence of preterm birth is high (18.1%) in Malawi, and inadequate maternal selenium status could be a contributing factor (10). Inadequate selenium intake is endemic in Malawi, where soil and staple crops have low selenium content (11). It is estimated that >80% of the Malawian population is at risk of dietary selenium inadequacy (11). In a nationally representative sample of women of reproductive age in 2015–2016, the prevalence of low plasma selenium concentrations (defined as <1.13 µmol/L) was 62.5% (12). In an earlier smaller study, 83% of pregnant women had low serum selenium concentrations (defined as <1 µmol/L) at 24 wk of gestation (13). Selenium supplementation during pregnancy can be an effective strategy for increasing maternal status. Supplementation with selenium-enriched yeast increased whole blood selenium concentrations in pregnant UK women with low selenium concentrations at enrollment (12–14 wk of gestation) (14). More information is needed on the effect of selenium supplementation during pregnancy on maternal status in populations known to be at risk of deficiency. This information would be useful for planning future studies to assess whether provision of selenium during pregnancy can be effective for preventing preterm birth in areas at high risk of selenium deficiency.

The International Lipid-based Nutrient Supplement (iLiNS) Project tested the efficacy of small-quantity lipid-based nutrient supplements (SQ-LNSs) in several trials, including a randomized, controlled, community-based trial in Malawi (iLiNS/DYAD-M) that was designed to assess the effect of providing SQ-LNSs to mothers during pregnancy and lactation, and to their children from 6 to 18 mo of age, on pregnancy outcomes, child growth and development, and other nutritional and health outcomes (https://ilins.ucdavis.edu/) (15, 16). Because the SQ-LNSs that were provided to women during pregnancy contained selenium, and there was emerging evidence that low selenium status during pregnancy is a risk factor for preterm birth (8), we conducted a subanalysis of the iLiNS/DYAD-M trial to assess whether provision of SQ-LNSs to Malawian women during pregnancy had an effect on their plasma selenium concentrations at 36 wk of gestation.

SQ-LNSs are food supplements that contain peanut paste, vegetable oil, and other ingredients (such as skim milk powder) that are enriched with vitamins and minerals to meet the needs of specific target populations. They were developed to prevent undernutrition in pregnant and lactating women, and in young children in low and middle-income countries (17). SQ-LNSs contain energy, protein, essential fatty acids, and a greater number of micronutrients than multiple micronutrients (MMN), including several macrominerals that are required for growth (17). Recent meta-analyses of SQ-LNSs demonstrated reductions in adverse birth outcomes (18) and in child stunting, wasting, impaired development, anemia, and mortality (19–23), but did not examine maternal selenium status.

## Methods

### Participants and study design

The iLiNS/DYAD-M trial was conducted in rural areas of the Mangochi District in southern Malawi; enrollment of pregnant women occurred between February 2011 and August 2012. The primary objective of the trial was to determine whether provision of SQ-LNSs to women during pregnancy and the first 6 mo of lactation, and to children from 6 to 18 mo of age, improves fetal and child growth to a greater extent than consumption of iron and folic acid (IFA) during pregnancy only, or an MMN capsule during pregnancy and the first 6 mo of lactation. The main results of the trial can be found elsewhere (16). Pregnant women who participated in the iLiNS/DYAD-M trial were included in this subanalysis if they had results for plasma selenium concentration at ≤20 wk of gestation and at 36 wk of gestation.

Women who visited the study clinics for antenatal care were eligible for the trial if they were ≤20 wk of gestation (confirmed by ultrasound) and >15 y of age. Exclusion criteria were milk or peanut allergy, chronic disease requiring medical attention, pregnancy complications (moderate to severe edema, hemoglobin <50 g/L, systolic blood pressure >160 mmHg, or diastolic blood pressure >100 mmHg), intent to move away from the area, not a resident of the area, or unwillingness to take the study supplement.

Informed consent was obtained from participants, and the study protocol was approved by the Institutional Review Board of the University of California, Davis, and the Ethics Committees of the College of Medicine, University of Malawi; and the Pirkanmaa Hospital District, Finland.

Pregnant women were randomly assigned to 1 of 3 groups to receive either: *1*) IFA (60 mg iron and 400 µg folic acid as a capsule; standard of care); *2*) MMN (130 µg selenium plus 17 micronutrients including 20 mg iron as a capsule); or *3*) SQ-LNS (20 g sachet that contained 130 µg selenium plus the same 17 micronutrients as the MMN capsule and 4 additional minerals: calcium, phosphorus, potassium, and magnesium). Briefly, a staff member who was not involved with data collection created individual randomization slips, in blocks of 9, and put them in sealed, numbered, opaque envelopes that were stored in numerical order. Eligible women were asked to choose 1 of the top 6 envelopes from the stack; the randomization slip inside the chosen envelope indicated her participant number and group assignment (15). The IFA and MMN capsules were identical in appearance. Fieldworkers delivered a 2-wk supply of participants’ assigned capsule or SQ-LNS to their homes every 14 d. Field workers told women in the IFA and MMN groups to consume 1 capsule/d with a glass of water; women in the SQ-LNS group were told to consume 1 sachet/d of SQ-LNS, mixed with a small amount of any food (16). Adherence was assessed by maternal report and by counting any unused capsules or SQ-LNS sachets from the previous 2-wk period. The field coordinator and field workers who delivered supplements to participants were aware of the participants’ group assignments; all remaining staff were masked to group assignments.

The IFA and MNN capsules were manufactured by DSM Nutritional Products South Africa (Pty) Ltd. The SQ-LNS was produced and packaged by Nutriset S.A.S. Capsules and SQ-LNSs were stored at 20–40°C at the field site. The MMN capsules and 20-g SQ-LNS contained 130 µg selenium in the form of sodium selenite. This amount of selenium was based on twice the amount that is added to the UNIMMAP (United Nations International Multiple Micronutrient Antenatal Preparation) formulation (24). We chose this amount (17) because 130 µg/d resulted in better pregnancy outcomes in women in Guinea-Bissau compared with a daily dose of 65 µg, and because it was known that pregnant women in Malawi are at risk of selenium deficiency (13) and would likely need more than the RDA for selenium for plasma concentrations to increase to adequate levels. Although this amount is ∼2.2 times higher than the US RDA for pregnant women (60 µg/d), it is below the tolerable upper level of intake (UL) of 400 µg/d (25). The nutrient compositions of the IFA, MMN, and SQ-LNS are shown in **Supplemental Table 1**.

### Collection of blood samples

Nonfasting blood samples were collected from women at ≤20 wk of gestation and at 36 wk of gestation. Time of day and time of consumption of last food other than tea or water were recorded. Blood was centrifuged to obtain plasma (2000 × *g* for 15 min at ambient temperature), which was aliquoted into cryovials and stored at −80°C for later analysis of the selenium concentration. Plasma samples were shipped in dry ice to the University of California, Davis (Davis, CA) and stored at −80°C, until shipped in dry ice to Cincinnati Children's Hospital Medical Center (Cincinnati, OH) for measurement of the selenium concentration.

### Laboratory analyses

Plasma selenium concentration was measured by inductively coupled mass spectrometry (ICP-MS; Agilent 7700, Agilent Technologies, Inc) as described previously (8). Baseline and endline plasma samples were analyzed separately. A cutoff value of <1 µmol/L was used to define inadequate selenium status (13).

Plasma concentrations of C-reactive protein (CRP) and α_1_-acid glycoprotein (AGP) were measured by immunoassay using a COBAS Integra Analyzer (Roche Diagnostics). Cutoff values for plasma markers of inflammation were CRP >5 mg/L and AGP >1 g/L.

### Statistical analysis

To maximize our ability to detect a difference in plasma selenium concentration among groups, and potential effect modification by preselected participant characteristics, we measured plasma selenium concentration in all women with paired samples at ≤20 wk of gestation and at 36 wk of gestation (*n *= 755). A statistical analysis plan was developed and posted prior to analysis, available at https://ilins.ucdavis.edu and in the **Supplemental Materials**. Distributions of outcome variables and key baseline variables were inspected for normality and transformed to natural logs, as necessary. Analyses were performed using SAS version 9.4 (SAS Institute Inc). The covariates included in the ANCOVA models were obtained from the scientific literature and were preselected in our analysis plan. Covariates were included in the model if they were associated with the outcome variable (*P* < 0.1) to reduce the uncertainty in intervention effect estimates, as recommended by CONSORT guidelines (http://www.consort-statement.org) and best practice guidance (26). Potential covariates for plasma selenium analyses included maternal estimated prepregnancy BMI (27), maternal education, primiparity, site of enrollment, season at enrollment, baseline household food insecurity score, baseline asset index, baseline plasma selenium concentration, and baseline HIV status. Interactions were examined between group assignment and preselected potential effect modifiers: baseline plasma selenium concentration, baseline HIV status, and baseline inflammation (plasma CRP >5 mg/L or AGP >1 g/L). For effect modification testing statistical inference is based on the full model *P*-interaction rather than analyses conducted separately within subgroups. If a statistically significant interaction (*P *< 0.1) was found then group means were examined at different levels of the predictor variable, either by category for categorical effect modifiers, or at selected percentile cutoffs for continuous variables to better understand the nature of the effect modification, as recommended by CONSORT guidelines and best practice guidance (26).

The primary statistical analysis was done by complete-case intention-to-treat. The difference in mean plasma selenium concentrations at 36 wk of gestation among the 3 intervention groups was tested with ANOVA (model without covariates) and ANCOVA (model with covariates) and *P *< 0.05 to indicate statistical significance. Post hoc pairwise comparisons of the intervention groups were done with the Tukey–Kramer test for ANOVA and ANCOVA if the global null hypothesis was rejected with *P *< 0.05.

The proportion of women with low plasma selenium concentrations at 36 wk of gestation was compared between intervention groups using logistic regression. Tukey–Kramer pairwise comparisons between groups were done in the context of logistic regression if the global null hypothesis was rejected with *P *< 0.05.

A per protocol analysis was performed that included only participants whose adherence was high, defined as reported consumption of supplements on ≥70% of supplementation days.

Plasma selenium concentration decreases during inflammation because albumin, which is one of the carrier proteins for selenium, is a negative acute-phase protein (28). We used correlation analysis to examine the relation between plasma selenium concentration and plasma concentrations of CRP and AGP, separately, at 36 wk of gestation. If there was a correlation (Spearman correlation *P *< 0.1) between markers of inflammation and plasma selenium concentration, the method described by the BRINDA (Biomarkers Reflecting Inflammation and Nutritional Determinants of Anemia) project was used to correct the selenium results for inflammation (29). Inflammation-corrected and non–inflammation-corrected plasma selenium results are presented.

Before applying the BRINDA correction, we first examined whether the intervention had a statistically significant effect on plasma concentrations of CRP or AGP at 36 wk of gestation.

## Results

### Participants

A total of 1391 women were enrolled in the iLiNS/DYAD-M trial. Of those, 755 had selenium results for paired plasma samples at ≤20 wk of gestation and 36 wk of gestation and were included in this subanalysis (**Supplemental Figure 1**). Enrollment characteristics for women that were excluded (*n *= 636) or included (*n *= 755) in the selenium subanalysis are shown in **Supplemental Table 2**. Women who were included differed from those who were excluded in that, on average, they were older (25.3 compared with 24.5 y), completed more years at school (4.2 compared with 3.8 y), and had a lower prevalence of anemia (17.7% compared with 24.4%), a lower prevalence of primiparity (18.7% compared with 25.8%), lower plasma selenium concentration (1.03 compared with 1.08 µmol/L), and a higher prevalence of low inflammation-corrected plasma selenium concentrations (40.1% compared with 33.9%).

Maternal characteristics at enrollment, by intervention group, are shown in **Table 1**. Mean maternal age was ∼25 y, 17.8% were anemic, 13.1% were HIV-positive, 22.4% tested positive for malaria, 41.7% had elevated plasma CRP concentrations, 13.8% had elevated plasma AGP concentrations, and 40.1% had inflammation-corrected plasma selenium concentrations <1.0 µmol/L.

**TABLE 1:** Characteristics of participants by group at enrollment^1^

Characteristic	IFA	MMN	SQ-LNS
Number of participants	245	261	249
BMI, kg/m^2^	21.8 ± 2.5	21.9 ± 3.0	21.8 ± 2.8
Maternal age, y	25.1 ± 5.9	25.6 ± 6.1	25.2 ± 6.1
Maternal education (completed years at school)	4.2 ± 3.4	4.2 ± 3.3	4.2 ± 3.5
Proxy for SES	0.01 ± 1.05	−0.05 ± 0.96	0.01 ± 1.07
Anemia (Hb <100 g/L), %	18.4	17.6	17.3
Primiparous, %	18.9	17.2	20.1
Low BMI (<18.5 kg/m^2^), %	6.2	8.1	8.1
Positive HIV test, %	14.5	10.8	14.1
Positive malaria test (RDT), %	20.4	24.5	22.2
Plasma selenium concentration, µmol/L	1.03 ± 0.39	1.03 ± 0.37	1.04 ± 0.38
Plasma selenium concentration <1 µmol/L, %	56.1	54.5	52.3
Plasma CRP concentration >5 mg/L, %	38.5	37.8	48.8
Plasma AGP concentration >1 g/L, %	15.2	10.8	15.3
Inflammation-corrected plasma selenium concentration <1 µmol/L, %	40.6	41.5	38.2

1 Values are mean ± SD or percentage. AGP, α_1_-acid glycoprotein; CRP, C-reactive protein; Hb, hemoglobin; IFA, iron folic acid; MMN, multiple micronutrients; RDT, rapid diagnostic test; SES, socioeconomic status; SQ-LNS, small-quantity lipid-based nutrient supplement.

### Inflammation

At 36 wk of gestation, the overall prevalence of elevated CRP and AGP concentrations was 29.5% and 5%, respectively, and the prevalence did not differ by intervention group (*P *= 0.237 and *P *= 0.220, respectively).

### Plasma selenium concentrations at 36 wk of gestation

Unadjusted geometric mean (GM) plasma selenium concentrations tended to differ by group (*P *= 0.069; **Table 2**). After correcting for inflammation and adjusting for baseline plasma selenium concentration, season, and site, the geometric mean ratio (GMR) of plasma selenium concentrations differed significantly by intervention group (*P *= 0.007); it was higher in the SQ-LNS group than in the MMN group (GMR = 1.05; 95% CI: 1.02, 1.09; Table 2), and it tended to be higher in the SQ-LNS group than in the IFA group (GMR = 1.04; 95% CI: 1.01, 1.08; Table 2).

**TABLE 2:** Maternal plasma selenium concentration at 36 wk of gestation by intervention group^1^

	Geometric mean (95% CI)				Comparison between LNS and IFA group		Comparison between MMN and IFA group		Comparison between LNS and MMN group	
Outcome	IFA (*n *= 245)	MMN (*n *= 261)	LNS (*n *= 249)	*P* value	GMR (95% CI)	Tukey–Kramer *P* value	GMR (95% CI)	Tukey–Kramer *P* value	GMR (95% CI)	Tukey–Kramer *P* value
Plasma selenium,^2^ µmol/L	0.92 (0.88, 0.96)	0.93 (0.89, 0.97)	0.98 (0.94, 1.03)	0.069	1.07 (1.00, 1.14)	0.093	1.01 (0.95, 1.07)	0.978	1.06 (1.00, 1.13)	0.133
Inflammation-corrected plasma selenium,^3^µmol/L	0.97 (0.95, 1.00)	0.96 (0.94, 0.99)	1.02 (0.99, 1.04)	0.007	1.04 (1.01, 1.08)	0.053	0.99 (0.96, 1.02)	0.783	1.05 (1.02, 1.09)	0.007

1 GMR, geometric mean ratio; IFA, iron folic acid; LNS, lipid-based nutrient supplement; MMN, multiple micronutrients

2 Unadjusted value.

3 Adjusted for baseline plasma selenium concentration, season and study site.

The prevalence of unadjusted plasma selenium concentrations <1 µmol/L did not differ by group (**Table 3**). After correcting for inflammation and adjusting for baseline plasma selenium concentration and season, the prevalence of low values differed by group (*P *= 0.007; Table 3); it was lower in the SQ-LNS group than in the MMN group (OR = 0.44; 95% CI: 0.24, 0.83), and it tended to be lower in the SQ-LNS group than in the IFA group (OR = 0.54; 95% CI: 0.29, 1.03).

**TABLE 3:** Prevalence of low maternal plasma selenium concentration at 36 wk of gestation by intervention group^1^

	Prevalence				Comparison between LNS and IFA group		Comparison between MMN and IFA group		Comparison between LNS and MMN group	
Outcome	IFA (*n *= 245)	MMN (*n *= 261)	LNS (*n *= 249)	*P* value	OR (95% CI)	Tukey–Kramer *P* value	OR (95% CI)	Tukey–Kramer *P* value	OR (95% CI)	Tukey–Kramer *P* value
Plasma selenium <1.0 µmol/L,^2^ %	60.0	62.8	55.0	0.194	0.82 (0.53, 1.25)	0.503	1.13 (0.73, 1.73)	0.790	0.72 (0.47, 1.11)	0.173
Inflammation-corrected plasma selenium <1.0 µmol/L,^3^ %	55.1	57.8	47.3	0.007	0.55 (0.29, 1.03)	0.063	1.23 (0.66, 2.30)	0.708	0.44 (0.24, 0.83)	0.006

1 IFA, iron folic acid; LNS, lipid-based nutrient supplement; MMN, multiple micronutrients.

2 Unadjusted values.

3 Adjusted for baseline selenium concentration and season.

**FIGURE 1:**
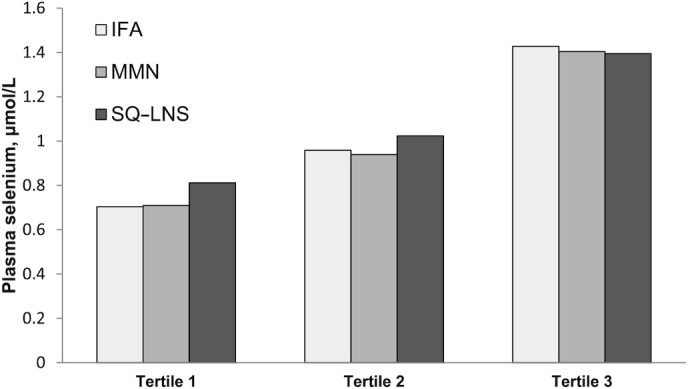
Geometric mean inflammation-corrected selenium concentration at 36 wk of gestation stratified by tertile of baseline plasma selenium concentration. Results are corrected for inflammation and adjusted for baseline plasma selenium concentration; *P*-interaction = 0.005. GM plasma selenium concentration was higher in the SQ-LNS group compared with the MMN and IFA groups in women in the lowest tertile (GMR = 1.14; 95% CI: 1.07, 1.23; and GMR = 1.15; 95% CI: 1.07, 1.24). In the middle tertile, it was higher in the SQ-LNS group than in the MMN group (GMR = 1.09; 95% CI: 1.01, 1.17) and tended to be higher than in the IFA group (GMR = 1.07; 95% CI: 0.99, 1.15). The GM values did not differ by group in the upper tertile. GM, geometric mean; GMR, geometric mean ratio; IFA, iron folic acid; MMN, multiple micronutrients; SQ-LNS, small-quantity lipid-based nutrient supplement.

**FIGURE 2:**
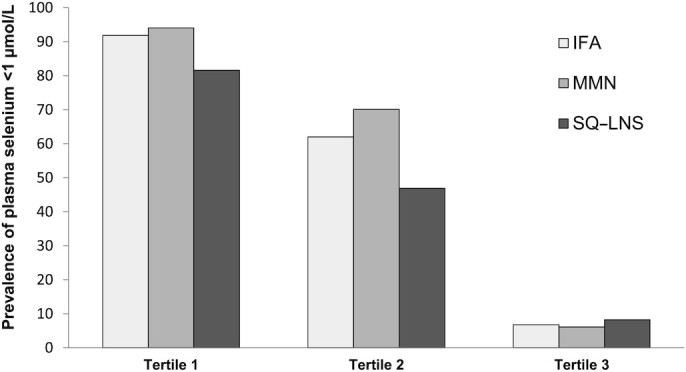
Prevalence of plasma selenium concentrations <1 µmol/L stratified by tertile of baseline plasma selenium concentration. Results are corrected for inflammation and adjusted for baseline plasma selenium concentration; *P*-interaction = 0.003. Prevalence of low plasma selenium concentration was lower in the SQ-LNS group than in the MMN and IFA groups in the lowest tertile (OR = 0.39; 95% CI: 0.15, 1.00; and OR = 0.28; 95% CI: 0.10, 0.81). In the middle tertile, it was lower in the SQ-LNS group than in the MMN group (OR = 0.38; 95% CI: 0.20, 0.71), but did not differ from that of the IFA group. There were no differences by group in the upper tertile. IFA, iron folic acid; MMN, multiple micronutrients; SQ-LNS, small-quantity lipid-based nutrient supplement.

There was a significant interaction between baseline plasma selenium concentration and intervention group for GM plasma selenium concentration (*P*-interaction = 0.005, **Figure 1**) and the prevalence of low plasma selenium concentrations at 36 wk of gestation (*P*-interaction = 0.003; **Figure 2**). The effect of SQ-LNS on plasma selenium concentration was greater in women whose baseline plasma selenium concentration was in the lowest or middle tertiles, and was not evident in those with a baseline selenium concentration in the upper tertile. There were no other interactions between the preselected potential effect modifiers and intervention group.

### Per protocol analysis

High adherence was reported by 199 (81.2%) women in the IFA group, 220 (84.3%) in the MMN group, and 213 (85.5%) in the SQ-LNS group. Based on the per protocol analysis, GM plasma selenium concentration differed by group at 36 wk of gestation after correcting for inflammation and adjusting for baseline plasma selenium concentration, season, and site (*P *< 0.01). GM plasma selenium concentration was higher in the SQ-LNS group compared with the MMN group (GMR = 1.06; 95% CI: 1.02, 1.10), and it tended to be higher in the SQ-LNS group than in the IFA group (GMR = 1.04; 95% CI: 1.00, 1.08). The prevalence of low plasma selenium concentrations at 36 wk of gestation tended to differ by group after correcting for inflammation and adjusting for baseline plasma selenium concentration, mother's education, and season (*P *= 0.071). The prevalence of low values tended to be lower in the SQ-LNS group than in the MMN group (OR = 0.52; 95% CI: 0.26, 1.04). There were no other differences by group.

## Discussion

In this study, we provided SQ-LNSs containing selenium to women during pregnancy and assessed the effect of SQ-LNSs on maternal plasma selenium concentrations at 36 wk of gestation. Provision of SQ-LNSs from ≤20 wk to 36 wk of gestation increased plasma selenium concentrations and reduced the prevalence of low plasma selenium concentrations in women with low plasma selenium concentrations at baseline.

At ≤20 wk of gestation the overall prevalence of inflammation-corrected low plasma selenium concentrations (<1 µmol/L) was ∼40%. The high prevalence of inadequate status is not surprising given that soil and staple crops have low selenium content in Malawi, and the population is known to be at risk of inadequate selenium intake (30). Although there was a significant reduction in the prevalence of low selenium concentrations in women in the SQ-LNS group compared with the other groups, the prevalence of low values remained high (∼47.3%). We used a cutoff value of <1 µmol/L for inadequate selenium status, which might overestimate the prevalence of selenium deficiency at 36 wk of gestation because of hemodilution that occurs during pregnancy. A decline in plasma selenium concentrations during pregnancy has been observed in European women with low concentrations in early pregnancy (31, 32). Plasma selenium concentrations were lower in the third trimester than in the first trimester, and this is likely related to increased transfer of selenium to the fetus in the third trimester, when the fetal liver is able to store selenium, and/or hemodilution (31). In contrast, in women with adequate selenium status, plasma selenium concentrations appear to remain unchanged during pregnancy. The mean plasma selenium concentration was similar in pregnant Finnish women with adequate status during the first, second, and third trimesters (1.34 ± 0.19 µmol/L, 1.36 ± 0.18 µmol/L, and 1.37 ± 0.19 µmol/L, respectively). Although none of the women had low plasma concentrations during pregnancy their plasma selenium concentrations increased after delivery (33). In US women, the mean plasma selenium concentration was ∼1.9 µmol/L throughout pregnancy (34). More research is needed to understand the extent to which plasma selenium concentrations are affected by hemodilution, and which cutoff values would be most appropriate in the first, second, and third trimesters of pregnancy.

Nutritional strategies for improving selenium status include supplementation, food fortification, point-of-use fortification, and promotion of production and consumption of foods that are naturally high in selenium content, where possible. In this study, we examined the effect of SQ-LNSs as a point-of-use fortification strategy for improving selenium status during pregnancy. This approach allows for direct targeting of populations that are likely to benefit from the intervention, such as pregnant women, and the amounts of nutrients that are added to the food vehicle can be formulated to meet the specific needs of the target population. Both the SQ-LNS and MMN that were provided to women in our study contained 130 µg selenium; however, plasma selenium concentration increased only in women who were provided with the SQ-LNS. The reason for this is unknown, but it could be related to a difference in bioavailability of selenium when it is consumed within a food-matrix (SQ-LNS) compared with as an MMN. Selenium is absorbed efficiently, but retention of organic forms of selenium (selenomethionine and other selenoproteins) is higher than that of inorganic selenium (selenite, selenite) (35, 36). Both SQ-LNS and MMN contained selenium in the form of sodium selenite. The plasma response to supplementation with selenite is limited because it can be incorporated only into plasma selenoproteins. When plasma selenoprotein concentrations become saturated, plasma selenium concentrations plateau and continued supplementation no longer has an effect on plasma concentrations (37). This does not explain the difference in the plasma selenium response between the SQ-LNS and MMN groups, but it is possible that there is a difference in bioavailability of selenite from SQ-LNS compared with MMN. Bioavailability of selenium is difficult to quantify because of challenges in measuring the different forms of selenium in foods, and variable changes in indicators of selenium status in response to supplementation with selenium. Nevertheless, selenium bioavailability from food is enhanced when consumed with vitamins A, E, and D, and/or low-molecular-weight proteins containing methionine (36). SQ-LNS provides selenium in a food-matrix that contains vitamins and protein, whereas MMN provides selenium mixed with other micronutrients in a capsule. This might explain why plasma selenium concentrations responded to consumption of SQ-LNS but not to consumption of MMN. It is also possible that interactions between selenium and minerals in MMN, in the absence of a food matrix, reduced the bioavailability of selenium. Heavy metals are known to reduce bioavailability of selenium (36), but there is little information on whether mineral nutrients can reduce bioavailability. Reported adherence to supplements was high (∼84%), and similar in all groups, so lower adherence does not explain the lack of response in the MMN group.

In the United Kingdom, selenium status increased in pregnant women who were supplemented with selenium-enriched yeast (60 µg/d in the form of selenomethionine) from ∼12 wk to 35 wk of gestation: in women in the lowest tertile of baseline selenium status, median whole blood selenium concentration at 35 wk of gestation was 1.87 µmol/L compared with 1.16 µmol/L in the control group (2). In contrast to our findings, provision of SQ-LNS containing selenium (75 µg/d in the form of sodium selenite) to lactating HIV-positive Malawian women, beginning at 2 or 6 wk postpartum and continuing until 24 wk postpartum, had no effect on their plasma or breastmilk selenium concentrations at 24 wk postpartum in a randomized controlled trial (38). The lower dose and/or differences in absorption/retention or utilization of selenium in HIV-positive individuals or during lactation (compared with pregnancy) might account for the different results.

Our results suggest that provision of SQ-LNS during pregnancy can be an effective strategy for improving selenium status of pregnant women in areas at risk of deficiency. Our study has several strengths, including the randomized controlled study design, a sufficient sample size to detect differences in selenium status by intervention group, reported adherence to supplements, and accurate measurement of plasma selenium concentration by ICP-MS. Limitations to our study include lack of generalizability of results to the full study group because of differences at enrollment between women who were excluded compared with included in the plasma selenium subanalysis: the latter were slightly older, completed more years at school, and had a lower prevalence of anemia and primiparity and lower plasma selenium concentrations. Because women who were included in the analysis had a higher prevalence of low inflammation-corrected plasma selenium concentrations (40.1% compared with 33.9%) at enrollment, compared with those who were excluded, they might have been more responsive to the intervention. However, more than one-third of the excluded women also had low selenium status, indicating that it was common in the full study group. Other limitations include lack of data on dietary intake of selenium, the use of a single marker of selenium status, and uncertainty regarding an appropriate cutoff for plasma selenium concentration in late pregnancy. The amount of selenium provided in our study (130 µg/d) is ∼2.2 times the North American RDA for pregnant women but is much lower than the UL of 400 µg/d. Although our study population responded to SQ-LNS containing 130 µg/d, the prevalence of low plasma selenium concentrations remained high at 36 wk of gestation. More research is needed to determine an appropriate cutoff value for plasma selenium concentration in late pregnancy.

In conclusion, populations with a known risk of deficiency can benefit from a more bioavailable form of selenium or from SQ-LNS containing a larger amount of selenium in the form of sodium selenite. Because selenium deficiency is a risk factor for preterm birth, provision of SQ-LNS containing selenium during pregnancy should be evaluated as a strategy for reducing risk of preterm birth in areas at high risk of deficiency.

## Supplementary Material

nzac013_Supplemental_File

## Data Availability

Data described in the manuscript, codebook, and analytic code will be made available upon request pending application to the authors.

## Notes

Financial support for this work was provided by the Bill & Melinda Gates Foundation (Project Number OPP49817). The source of support had no role in the design or implementation of the study, or in data analysis or interpretation.

Author disclosures: The authors report no conflicts of interest.

Supplemental Figure 1, Supplemental Tables 1–2, and Supplemental Materials are available from the “Supplementary data” link in the online posting of the article and from the same link in the online table of contents at https://academic.oup.com/cdn/.

Abbreviations used: AGP, α_1_-acid glycoprotein; BRINDA, Biomarkers Reflecting Inflammation and Nutritional Determinants of Anemia; CRP, C-reactive protein; GM, geometric mean; GMR, geometric mean ratio; ICP-MS, inductively coupled plasma mass spectrometry; IFA, iron and folic acid; iLiNS, International Lipid-based Nutrient Supplement (Project); MMN, multiple micronutrients; SQ-LNS, small-quantity lipid-based nutrient supplement; UL, tolerable upper intake level.
