# Supplementary material for: Provision of Small-Quantity Lipid-Based Nutrient Supplements Increases Plasma Selenium Concentration in Pregnant Women in Malawi: A Secondary Outcome of a Randomized Controlled Trial
Source: Curr Dev Nutr. 2022 Mar 7;6(3):nzac013. doi: 10.1093/cdn/nzac013 (PMC8929992; doi:10.1093/cdn/nzac013)
Supplement: nzac013_Supplemental_File [file nzac013_supplemental_file.docx]

| Provision of small-quantity lipid-based nutrient supplements increases plasma selenium concentration among pregnant women in Malawi: a secondary outcome of a randomized controlled trial. Marjorie J Haskell. Online Supplementary Material |
| --- |

| Nutrient | IFA | MMN | SQ-LNS |  |  |
| --- | --- | --- | --- | --- | --- |
| Ration, g/d | 1 capsule | 1 capsule | 20 |  |  |
| Total energy, kcal |  |  | 118 |  |  |
| Protein, g |  |  | 2.6 |  |  |
| Fat, g |  |  | 10 |  |  |
| Linoleic acid, g |  |  | 4.59 |  |  |
| α-Linolenic acid, g |  |  | 0.59 |  |  |
| Vitamin A, *µ*g RE |  | 800 | 800 |  |  |
| Vitamin C, mg |  | 100 | 100 |  |  |
| Vitamin B-1, mg |  | 2.8 | 2.8 |  |  |
| Vitamin B-2, mg |  | 2.8 | 2.8 |  |  |
| Niacin, mg |  | 36 | 36 |  |  |
| Folic acid, µg | 400 | 400 | 400 |  |  |
| Pantothenic acid, mg |  | 7 | 7 |  |  |
| Vitamin B-6, mg |  | 3.8 | 3.8 |  |  |
| Vitamin B-12, *µ*g |  | 5.2 | 5.2 |  |  |
| Vitamin D, IU |  | 400 | 400 |  |  |
| Vitamin E, mg |  | 20 | 20 |  |  |
| Vitamin K, *µ*g |  | 45 | 45 |  |  |
| Iron, mg | 60 | 20 | 20 |  |  |
| Zinc, mg |  | 30 | 30 |  |  |
| Copper, mg |  | 4 | 4 |  |  |
| Calcium, mg |  |  | 280 |  |  |
| Phosphorus, mg |  |  | 190 |  |  |
| Potassium, mg |  |  | 200 |  |  |
| Magnesium, mg |  |  | 65 |  |  |
| Selenium, *µ*g |  | 130 | 130 |  |  |
| Iodine, *µ*g |  | 250 | 250 |  |  |
| Manganese, mg |  | 2.6 | 2.6 |  |  |
| IFA, iron folic acid; MMN, multiple micronutrient; SQ-LNS, small-quantity lipid-based nutrient supplements; ^1^ nutrient composition includes nutrients from ingredients and  from the premix. | | | | | |

Supplemental Table 1. Composition of supplements provided to pregnant women.

| Supplemental Table 2. Characteristics of participants at enrollment by inclusion in analysis ^1^. | | | |
| --- | --- | --- | --- |
| Characteristic | Excluded | Included | P-value |
| Number of participants | 636 | 755 |  |
| BMI, kg/m^2^ | 21.7 (2.6) | 21.8 (2.8) | 0.411 |
| Maternal age, years | 24.5 (6.2) | 25.3 (6.0) | 0.019 |
| Maternal education (completed years at school) | 3.8 (3.5) | 4.2 (3.4) | 0.030 |
| Proxy for SES | 0.01 (0.96) | -0.01 (1.02) | 0.694 |
| Anemia (Hb <100 g/L), % | 24.4 | 17.7 | 0.002 |
| Primiparous, % | 25.8 | 18.7 | 0.001 |
| Low BMI (<18.5 kg/m^2^), % | 6.5 | 7.5 | 0.461 |
| Positive HIV test, % | 14.0 | 13.1 | 0.632 |
| Positive malaria test (RDT), % | 24.2 | 22.4 | 0.427 |
| Plasma selenium concentration, µmol/L | 1.08 (0.38) | 1.03 (0.38) | 0.034 |
| Plasma selenium concentration <1 µmol/L, % | 47.3 | 54.3 | 0.015 |
| Plasma CRP concentration >5 mg/L, % | 41.8 | 41.7 | 0.959 |
| Plasma AGP concentration >1 g/L, % | 11.5 | 13.7 | 0.223 |
| Inflammation-corrected plasma selenium  concentration <1 µmol/L, % | 33.9 | 40.1 | 0.025 |

Values are mean (SD) or percent. RDT, rapid diagnostic test; CRP, C-reactive protein; AGP, α_1_-acid glycoprotein.^1^ Women were included in the sub-analysis if they had paired baseline/endline plasma samples for measurement of the selenium concentration. Women who were missing a blood sample at baseline and/or endline, or had insufficient plasma at either time point were excluded from the sub-analysis.

#

#
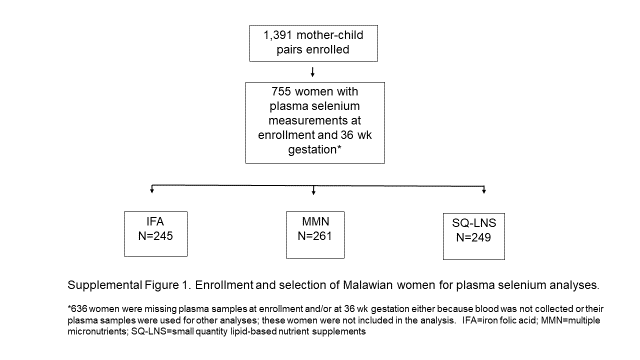


# Efficacy of Lipid-based Nutrient Supplements (LNS) for Pregnant and Lactating Women

# (iLiNS-DYAD-M)

**Statistical Analysis Plan**

Appendix 33b: Impact of LNS on maternal plasma selenium concentrations

Version 01.0, 2019-04-18

**Contents**

[Version History Log 2](#_Toc520982147)

[1. Background 3](#_Toc520982148)

[2. Study objectives 3](#_Toc520982150)

[3. Hypotheses 3](#_Toc520982151)

[4. Outcome measures 3](#_Toc520982153)

[5. Basis for the analysis: Intention to treat 3](#_Toc520982155)

[6. Time points 4](#_Toc520982156)

[7. Statistics software 4](#_Toc520982157)

[8. Outliers 4](#_Toc520982158)

[9. Data transformation 4](#_Toc520982159)

[10. Inflammation correction 4](#_Toc520982160)

[11. Covariates and effect modifiers 4](#_Toc520982163)

[12. Results 5](#_Toc520982165)

[Table 1. 6](#_Toc520982166)

[Table 2 7](#_Toc520982167)

[13. References 8](#_Toc520982170)

# Version History Log

| **Version number** | **Version date** | **Prepared by** | **Description of the completed editions** |
| --- | --- | --- | --- |
| 01.0 | 2019-04-18 | Marjorie Haskell | Original document appendix added |

#

# 1. Background

# The primary objective for the iLiNS-DYAD-M trial is to determine whether a small-quantity lipid-based nutrient supplement (LNS) consumed by women during pregnancy and the first 6 mo of lactation, and by the child from 6-18 mo of age, improves fetal and child growth, micronutrient status and neuro-behavioral development to a greater extent than consumption of iron and folic acid during pregnancy only, or a multiple micronutrient (MMN) tablet during pregnancy and the first six months of lactation. This analysis will examine the effect of LNS and MMN (both of which contained selenium) on the selenium status of women who participated in the main trial.

# 2. Study objectives

The objective for the present analyses is to determine the effect of LNS and MMN on maternal plasma selenium concentration. Specifically:

1) To determine if there are differences in mean maternal plasma selenium concentrations at 36 wk gestation among women who received LNS or multiple micronutrient capsules (MMN) during pregnancy, compared to women who received iron and folic acid capsules (IFA) during pregnancy.

2) To determine if differences exist in the prevalence of low plasma selenium concentrations at 36 wk gestation among women who received LNS or multiple micronutrient capsules (MMN) during pregnancy, compared to women who received iron and folic acid capsules (IFA) during pregnancy.

3) To examine whether plasma selenium concentration at ≤20 wk gestation is associated with plasma selenium concentration at 36 wk gestation, controlling for intervention group.

**3. Hypotheses**

1) Women who received LNS or MMN during pregnancy will have a higher mean plasma selenium concentration at 36 wk gestation compared to the IFA group.

2) Women who received LNS or MMN during pregnancy will have a lower prevalence of low selenium concentrations at 36 wk gestation compared to the IFA group.

3) Plasma selenium concentrations at ≤20 wk gestation will be positively associated with plasma selenium concentrations at 36 wk gestation, controlling for intervention group.

# 4. Outcome measures

# 1) Plasma selenium concentration (primary outcome measure)

a. Plasma selenium concentration will be analyzed as a continuous outcome.

b. Low plasma selenium concentration will be defined as <1.0 µmol/L (there is no pregnancy-specific cut-off for plasma selenium concentration).

#

# 5. Basis for the analysis: Intention to treat

The primary analysis will be by complete case intention-to-treat. Data on participants who were lost to follow-up because of death, travel from the study site, or refusal to continue with the study will be included in the analysis if their data are available.

In addition to the intention to treat analysis, a per protocol analysis will be performed including subjects meeting minimum criteria for adherence to study protocol. Adherence is recorded biweekly by interview of study subject and verified by collection and count of remaining intervention supplements. Good adherence will be defined as consumption on ≥70% of supplement days and minimum adherence will be defined as consumption on > 50% of supplement days during pregnancy.

If no difference in plasma selenium concentration is observed between the LNS and MMN groups, a second sensitivity analysis will be conducted that combines these groups to assess whether there is a difference in plasma selenium concentration among women who received LNS or MMN and women who received IFA.

# 6. Time points

Blood samples were collected from women for measurement of plasma selenium concentration at ≤20 wk gestation and 36 wk gestation.

# 7. Statistics software

Analyses will be performed using SAS version 9.4.

# 8. Outliers

Outliers will be visually inspected by creating box and whisker plots and/or histograms of individual continuous variables, and scatterplots of related variables. Outliers which are clearly impossible or implausible values will be corrected if possible, or recoded to missing if correction is not possible. Outliers which are plausible or possible will be kept.

# 9. Data transformation

Distribution of outcome variables and key baseline variables will be inspected for normality and transformed as necessary. If no suitable transformation is found, normalized ranks will be calculated, or categories will be created.

# 10. Inflammation correction

# Plasma selenium concentrations may be altered during inflammation. To assess whether there is an association between plasma selenium concentrations and plasma markers of inflammation, correlation analysis will be used to examine the relationship between plasma selenium concentration and plasma concentrations of CRP and AGP, separately, at the 36 wk timepoint. If there is a statistically significant correlation (Spearman's correlation p<0.1) between markers of inflammation and plasma selenium concentration, regression analysis will be used to correct the plasma selenium results for inflammation. Both inflammation-corrected and non-corrected plasma selenium results will be presented.

# Before applying the inflammation correction, we will first examine whether the intervention had a statistically significant effect on plasma concentrations of CRP or AGP at the 36 wk timepoint. If there is an intervention effect on plasma concentrations of CRP or AGP, the beta-coefficient for the inflammation adjustment will be derived from the IFA group and applied to all groups, using regression analysis.

# 11. Covariates and effect modifiers

The covariates to be included in the ANCOVA models will be derived from the list below. Each variable that shows a statistically significant association with each outcome (P<0.1), will be included in the adjusted models.

Interactions will be examined between the intervention group and the variables listed below. If a statistically significant interaction (p<0.1) is found, group means will be examined at different levels of the predictor variable, either by category for categorical effect modifiers, or at selected percentile cutoffs for continuous variables. Variables to be examined as covariates include:

Covariates

1. Maternal prepregnancy BMI

1. Maternal education
2. Primiparity
3. Site of enrollment
4. Season at enrollment
5. Baseline household food insecurity score
6. Baseline asset index
7. Baseline (≤20 wk gestation) plasma selenium concentration
8. Baseline (≤20 wk gestation) HIV status

Effect Modifiers

1. Primiparity

2. Baseline (<20 wk gestation) plasma selenium concentration

3. Baseline (<20 wk gestation) HIV status

4. Baseline inflammation (plasma CRP >5 mg/L; AGP >1 g/L)

# 12. Results

Group means and standard deviations for maternal plasma selenium concentrations will be tabulated by intervention group and presented in Tables 1. The table will also indicate the differences in means and their 95% confidence intervals between the intervention groups.

The difference between the three groups will be tested with ANOVA (model without covariates) and ANCOVA (model with covariates) and null-hypothesis of no difference between groups will be rejected if P<0.05. Proc GLIMMIX in SAS will be used to run a linear regression model of continuous outcomes. If the null-hypothesis is rejected, post-hoc pairwise comparisons of the three intervention groups will be done with the Tukey-Kramer test for ANOVA. For all pairwise comparisons with P<0.05, the null-hypothesis of no difference in means between groups will be rejected.

The proportion of women with low plasma selenium concentrations will be tabulated by intervention group as shown in Tables 2. Proc Logistic in SAS will be used to run a logistic regression model of the binary outcomes. Global null hypothesis of no differences between groups will be tested. Pairwise comparisons between groups will be done in the context of logistic regression if global null- hypothesis is rejected with P<0.05. Odds ratios between intervention groups are also presented in Table 2.

| Table 1. Maternal plasma selenium concentration at 20 wk gestation and 36 wk gestation by intervention group. | | | | | | | | | | |
| --- | --- | --- | --- | --- | --- | --- | --- | --- | --- | --- |
|  | Result by study group | | | | Comparison between LNS and IFA group | | Comparison between MMN and IFA group | | Comparison between LNS and MMN group | |
| Outcome | IFA  (n=xxx) | MMN  (n=xxx) | LNS  (n-xxx) | p-value | Difference in means (95% CI) | p-value | Difference in means (95% CI) | p-value | Difference in means (95% CI) | p-value |
| Mean (SD) plasma selenium (µmol/L) at 20 wk gestation | x.xx  (x.xx) | x.xx  (x.xx) | x.xx  (x.xx) | 0.xxx | x.xx (x.xx,x.xx) | 0.xxx | x.xx (x.xx,x.xx) | 0.xxx | x.xx (x.xx,x.xx) | 0.xxx |
| Difference in mean, adjusted model | - | - | - | 0.xxx | x.xx (x.xx,x.xx) | 0.xxx | x.xx (x.xx,x.xx) | 0.xxx | x.xx (x.xx,x.xx) | 0.xxx |
| Inflammation-corrected mean (SD) plasma selenium (µmol/L) at 20 wk gestation | x.xx  (x.xx) | x.xx  (x.xx) | x.xx  (x.xx) | 0.xxx | x.xx (x.xx,x.xx) | 0.xxx | x.xx (x.xx,x.xx) | 0.xxx | x.xx (x.xx,x.xx) | 0.xxx |
| Difference in mean, adjusted model | - | - | - | 0.xxx | x.xx (x.xx,x.xx) | 0.xxx | x.xx (x.xx,x.xx) | 0.xxx | x.xx (x.xx,x.xx) | 0.xxx |
| Mean (SD) plasma selenium (µmol/L) at 36 wk gestation | x.xx  (x.xx) | x.xx  (x.xx) | x.xx  (x.xx) | 0.xxx | x.xx (x.xx,x.xx) | 0.xxx | x.xx (x.xx,x.xx) | 0.xxx | x.xx (x.xx,x.xx) | 0.xxx |
| Difference in mean, adjusted model | - | - | - | 0.xxx | x.xx (x.xx,x.xx) | 0.xxx | x.xx (x.xx,x.xx) | 0.xxx | x.xx (x.xx,x.xx) | 0.xxx |
| Inflammation-corrected mean (SD) plasma selenium (umol/L) at 36 wk gestation | x.xx  (x.xx) | x.xx  (x.xx) | x.xx  (x.xx) | 0.xxx | x.xx (x.xx,x.xx) | 0.xxx | x.xx (x.xx,x.xx) | 0.xxx | x.xx (x.xx,x.xx) | 0.xxx |
| Difference in mean, adjusted model | - | - | - | 0.xxx | x.xx (x.xx,x.xx) | 0.xxx | x.xx (x.xx,x.xx) | 0.xxx | x.xx (x.xx,x.xx) | 0.xxx |

| Table 2. Differences in proportions of women with low plasma selenium concentration at 20 wk gestation and 36 wk gestation by intervention group. | | | | | | | | | | |
| --- | --- | --- | --- | --- | --- | --- | --- | --- | --- | --- |
|  | Result by study group | | | | Comparison between LNS and IFA group | | Comparison between MMN and IFA group | | Comparison between LNS and MMN group | |
| Outcome | IFA  (n=xxx) | MMN  (n=xxx) | LNS  (n-xxx) | p-value | Odds ratio (95% CI) | p-value | Odds ratio (95% CI) | p-value | Odds ratio (95% CI) | p-value |
| Plasma selenium <1.0 µmol/L at 20 wk gestation | xx  (xx.x%) | xx  (xx.x%) | xx  (xx.x%) | 0.xxx | x.xx  (xx.x to xx.x) | 0.xxx | x.xx  (xx.x to xx.x) | 0.xxx | x.xx  (xx.x to xx.x) | 0.xxx |
| OR, adjusted model | - | - | - | 0.xxx | x.xx  (xx.x to xx.x) | 0.xxx | x.xx  (xx.x to xx.x) | 0.xxx | x.xx  (xx.x to xx.x) | 0.xxx |
| Inflammation-corrected plasma selenium <1.0 umol/L at 20 wk gestation) | xx  (xx.x%) | xx  (xx.x%) | xx  (xx.x%) | 0.xxx | x.xx  (xx.x to xx.x) | 0.xxx | x.xx  (xx.x to xx.x) | 0.xxx | x.xx  (xx.x to xx.x) | 0.xxx |
| OR, adjusted model | - | - | - | 0.xxx | x.xx  (xx.x to xx.x) | 0.xxx | x.xx  (xx.x to xx.x) | 0.xxx | x.xx  (xx.x to xx.x) | 0.xxx |
| Plasma selenium <1.0 µmol/L at 36 wk gestation | xx  (xx.x%) | xx  (xx.x%) | xx  (xx.x%) | 0.xxx | x.xx  (xx.x to xx.x) | 0.xxx | x.xx  (xx.x to xx.x) | 0.xxx | x.xx  (xx.x to xx.x) | 0.xxx |
| OR, adjusted model | - | - | - | 0.xxx | x.xx  (xx.x to xx.x) | 0.xxx | x.xx  (xx.x to xx.x) | 0.xxx | x.xx  (xx.x to xx.x) | 0.xxx |
| Inflammation-corrected plasma selenium <1.0 umol/L at 36 wk gestation | xx  (xx.x%) | xx  (xx.x%) | xx  (xx.x%) | 0.xxx | x.xx  (xx.x to xx.x) | 0.xxx | x.xx  (xx.x to xx.x) | 0.xxx | x.xx  (xx.x to xx.x) | 0.xxx |

**13. References**:

1. Hurst R, Siyame EW, Young SD, Chilimba ADC, Joy EJM, Black CR, Ander EL, Watts MJ, Chimima B, Gondwe J, Kang’ombe D, Stein AJ, Fairweather-Tait S, Gibson RS, Kalimbira AA, Broadley MR. Soil-type influences human selenium status and underlies widespread selenium deficiency risks in Malawi. Scientific Reports, 3:1425, 1-6, 2013.
2. Gibson RS, Bailey KB, Ampong Romano AB, Thomson CD. Plasma selenium concentrations in pregnant women in two countries with contrasting soil selenium levels. J Trace Elements Med Biol, 25, 230-235, 2011.
3. Diana A, Haszard JJ, Purnamasari DM, Nurulazmi I, Luftimas ED, Rahmania S, Nugraha GI, Erhardt J, Gibson RS, Houghton L. Iron, zinc, vitamin A and selenium status in a cohort of Indonesian infants after adjusting for inflammation using several different approaches. Br J Nutr, 118, 830-839, 2017
